# Surgical treatment of ipsilateral multi-level femoral fractures

**DOI:** 10.1186/s13018-014-0149-5

**Published:** 2015-01-24

**Authors:** Christian von Rüden, Markus Tauber, Alexander Woltmann, Jan Friederichs, Simon Hackl, Volker Bühren, Christian Hierholzer

**Affiliations:** Department of Trauma Surgery, Trauma Center Murnau, 82418 Murnau, Germany

**Keywords:** Ipsilateral multi-level femoral fracture, Rendezvous technique, All-in-one device, Long-term outcome

## Abstract

**Background:**

Concurrent ipsilateral fractures of the proximal and shaft of the femur are rare complex fracture combinations. In this prospective cohort study, we evaluated clinical and radiological long-term results after operative treatment using several surgical strategies: the so-called “rendezvous” surgical technique, e.g., the combination of retrograde intramedullary nailing and dynamic hip screw (DHS) osteosynthesis, or the all-in-one device technique, e.g., long cephalomedullary nail, compared with two non-overlapping implants (e.g., conventional technique).

**Methods:**

In a 10-year-period from 2004 to 2013, we treated 65 patients with complex ipsilateral multi-level femoral fractures. Median age was 45 years (range 19–90 years). Fractures were classified according to the AO/OTA classification. Four patients died during intensive care unit treatment due to multi-organ failure prior to definitive osteosynthesis. Clinical long-term outcome using the functional system of Friedman/Wyman as well as radiological outcome was evaluated 2 years after trauma (range 13–42 months).

**Results:**

All-in-one device was used in 36 patients, “rendezvous” technique in 9 patients, and the conventional technique in the remaining 16 patients. Two years after trauma, complete fracture healing was found in 57 out of 61 patients (“rendezvous”: 9, all-in-one device: 33, conventional: 15; *p*-value: 0.66). There was no significant difference regarding the complication rate in the cohort groups (“rendezvous”: 3, all-in-one device: 13, conventional: 5; *p*-value: 0.94). Using the functional assessment system of Friedman/Wyman 2 years after trauma, a good clinical result was found in 77.7% in the “rendezvous” group, in 77.8% in the all-in-one device group, and in 75% in the conventional group.

**Conclusion:**

The indication for operative stabilization of ipsilateral multi-level femoral fractures is considered an urgent and emergency procedure. Based on the successful long-term results of this study, we prefer the “rendezvous” technique with fracture stabilization from distally to proximally. Both fracture components require stable fixation. It is advisable to stabilize the shaft fracture primarily using external fixation (damage control orthopedics) and the proximal femoral fracture using early definitive internal fixation. In a second and staged operation, the external fixator is removed and the shaft fracture is stabilized using retrograde nail osteosynthesis with overlapping of the DHS and nail implants.

## Background

Concurrent ipsilateral fractures of the shaft and proximal femur are rare. In 1%–9% of all femoral shaft fractures, an additional proximal fracture of the femur is noted [1]. Predominately, male patients in the fourth decade of their life are suffering these fractures following polytraumatization [1]. The mechanism of injury is originating predominately from a high energy trauma in contrast to low energy trauma or pathological fractures. Alho et al. classified these combined fractures as “complex femoral fractures” [2]. In 25% of cases, open fractures are observed. The incidence of associated adjacent injuries is high. In 40% of patients, injury to the ipsilateral knee joint occurs. Interestingly, a high rate of missed proximal fractures has been reported ranging from 10%–30% [3,4]. Few studies exist in the literature and cohort groups are comparatively small. The biggest meta-analysis included 722 cases [3]. A variety of treatment concepts have been proposed. Haas et al. described 30 possible surgical treatment options [4]. Surgical stabilization of these complex fractures is technically challenging and associated with a significant rate of complications.

## Methods

From 2004 to 2013, 65 patients (47 male; 18 female) with ipsilateral proximal and shaft fractures of the femur were treated in the German Level I Trauma Center Murnau and a prospective cohort study was performed. Median age was 45 years ranging from 19 to 90 years. The mechanism of injury was predominately high energy injuries including car (26 patients) or motorbike accidents (26 patients) and fall from height (13 patients). Polytraumatization occurred in 57 patients, while a mono-trauma was observed in the remaining 8 patients. All patients, included in this study, suffered at least two independent fracture localizations in the ipsilateral femur. Four patients died during ICU treatment due to multi-organ failure prior to definitive osteosynthesis. Fractures were classified according to the AO/OTA classification (Table 1). Open fractures were observed in four patients, among them two type 2 open and two type 3 open fractures using the Gustilo/Anderson classification. The diagnosis of the proximal fracture was missed initially in two patients. Initial patient care was performed by strictly adhering to ATLS® guidelines. In the emergency department, full-body spiral CT scanning was performed to assess multi-organ injury. Thin slice imaging of the head, thorax, abdomen, and the pelvic region including axial, coronal, and sagittal reconstructions were obtained to evaluate injuries. This approach is beneficial in detecting non-displaced fractures of the proximal femur. CT scout imaging is analyzed for bony injuries of the lower limbs. In cases of positive fracture findings, additional conventional X-rays were performed including anterior-posterior (AP) and lateral views of the femur with adjacent joints if the patient was hemodynamically stable and no additional contra-indications from multi-organ injuries were found. In cases of monotrauma, conventional X-ray studies using AP and lateral views of the vertebral column, the thorax, the pelvis, and the affected limbs were ordered depending on the mechanism of injury and findings of a thorough physical examination including neuro-vascular evaluation. Following initial resuscitation and stabilization of vital organ functions, osteosynthesis of femoral fractures was addressed.

**Table 1:** **Fracture classification according to AO/OTA classification, osteosynthesis material and fracture healing**

**Patient**	**Gender**	**Age**	**AO proximal**	**AO distal**	**All-in-one**	**Conventional proximally**	**Conventional distally**	**“Rendezvous”**	**Bone healing**
1	Male	78	31 A3	33 A2		CN	RN		Yes
2	Male	51	31 A1	32 C1	X				Yes
3	Male	39	32 A3	33 A1	X				Yes
4	Male	58	32 B1	32 B1	X				Yes
5	Female	51	32 A2	33 A2	X				Yes
6	Male	20	32 A3	32 A3	X				Yes
7	Male	35	32 C1	32 C1	X				Yes
8	Male	45	31 A3	32 A2	X				Yes
9	Female	51	31 A1	32 B2	X				Yes
10	Female	23	32 A2	32 A2	X				Yes
11	Male	40	31 A3	32 B3	X				Yes
12	Male	59	31 B2	32 A2				X	Yes
13	Male	43	31 B2	32 A2				X	Yes
14	Male	56	31 B2	32 C3		DHS	LP		No
15	Female	28	32 C1	32 C1	X				Yes
16	Male	61	31 A1	32 A1	X				Yes
17	Female	82	31 A3	33 A2		THA	RN		Yes
18	Male	49	32 A2	32 B2	X				Yes
19	Male	58	32 C1	32 A3	X				Yes
20	Male	72	31 A1	33 C3		DHS	RN		Yes
21	Male	61	31 B2	32 B2				X	Yes
22	Female	28	31 B1	32 B3		screws	RN		Yes
23	Male	30	32 B2	33 A2	X				Yes
24	Male	62	31 B2	32 A1		DHS	RN		Yes
25	Male	53	31 A1	33 A2		CN	LP		Yes
26	Male	46	31 B1	33 C3		Screws	LP		Yes
27	Male	52	31 A1	32 A2				X	Yes
28	Male	49	31 A3	32 A2	X				Yes
29	Male	52	32 B3	33 B2	X				Yes
30	Female	58	31 A3	32 A1	X				THA
31	Female	58	31 A3	32 B3	X				No
32	Male	47	31 A1	32 C1	X				Yes
33	Male	54	31 B1	32 B1		Screws	RN		Yes
34	Female	29	31 B1	32 C1		Screws	AN		Yes
35	Male	20	32 B3	32 B2	X				Yes
36	Male	20	32 C1	32 C1	X				Yes
37	Male	61	31 A1	32 B2	X				Yes
38	Male	57	31 A3	32 A3	X				Yes
39	Male	41	31 A2	33 B2		AN	LP		Yes
40	Male	59	31 A1	32 A2				X	Yes
41	Female	19	31 B1	32 A2	X				Yes
42	Exitus								
43	Female	49	32 A2	32 A2	X				Yes
44	Exitus								
45	Exitus								
46	Male	49	31 B3	32 A1	X				No
47	Exitus								
48	Male	43	31 A2	32 B1		DHS	LP		Yes
49	Male	39	31 A3	32 A2	X				Yes
50	Female	48	31 B2	32 A3				X	Yes
51	Male	20	32 A1	32 A3	X				Yes
52	Male	28	31 B2	32 C3				X	Yes
53	Male	47	31 B2	32 A2	X				Yes
54	Male	38	32 B2	32 A2	X				Yes
55	Male	24	31 B2	32 B2				X	Yes
56	Male	24	31 A1	32 B2	X				Yes
57	Male	53	31 A2	33 C2		DHS	LP		Yes
58	Male	78	31 A2	32 C3				X	Yes
59	Female	90	31 B2	32 A1		THA	LP		Yes
60	Male	48	31 B2	32 B1		DHS	RN		Yes
61	Female	77	31 A3	32 A2		CN/cerclages	LP		Yes
62	Male	31	32 B1	33 A1	X				Yes
63	Female	52	31 B2	32 B1	X				Yes
64	Male	29	31 B3	32 B2	X				Yes
65	Male	46	32 A2	32 A2	X				Yes

*THA* total hip arthroplasty, *DHS* dynamic hip screw, *LP* locking plate, *AN* antegrade nail, *RN* retrograde nail, *CN* cephalomedullary nail.

Treatment options for operative stabilization of multi-level femur fractures included the so-called “rendezvous” technique using the combination of retrograde intramedullary (i.m.) nailing and dynamic hip screw (DHS) osteosynthesis, or all-in-one device using the long cephalomedullary nail, compared with two non-overlapping implants (e.g., conventional surgical technique).

**Figure 1:**
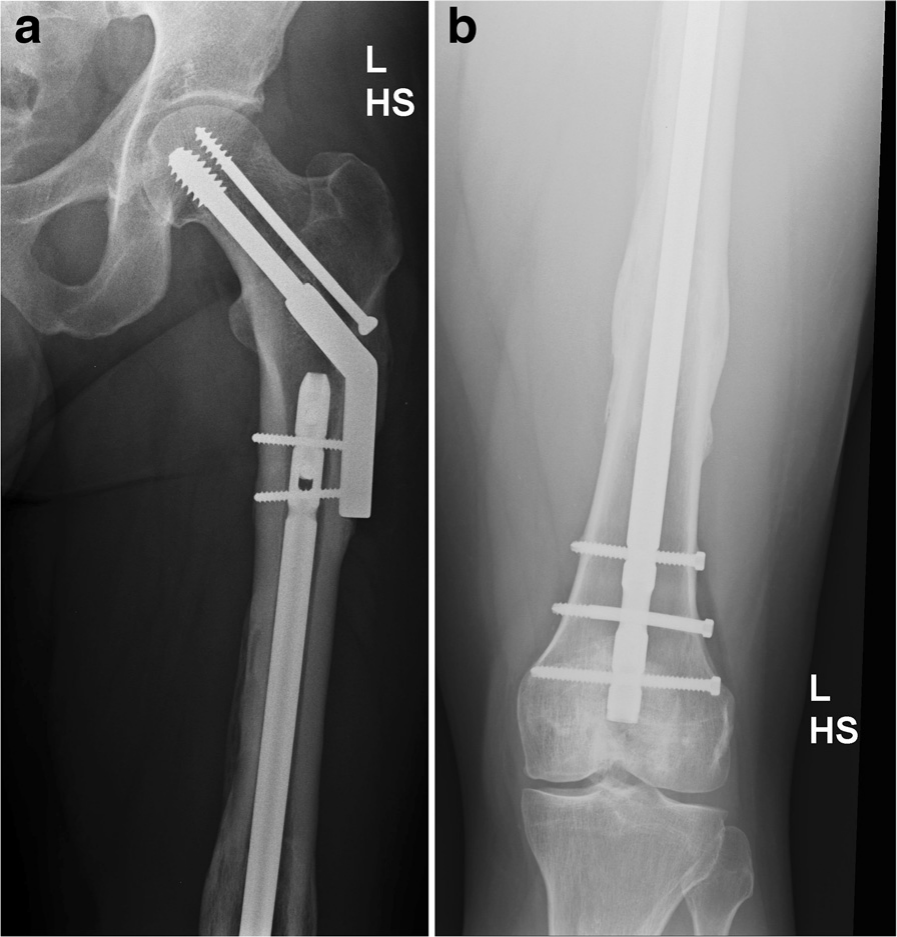
**38-year-old male patient after multiple trauma (femoral neck fracture and multi-fragment shaft fracture).** Two step stabilization, primarily with external fixator and DHS, secondarily with retrograde nailing using the “rendezvous” technique **(a, b)**.

The “rendezvous” technique offers the possibility to proceed with a two-step strategy and thereby following the principle of damage control orthopedics (DCO). On the day of injury, primary treatment of the shaft fracture is performed using external fixator stabilization, whereas the proximal fracture is stabilized using definitive DHS internal osteosynthesis. The second step, which is performed following stabilization of the general conditions of the patient after several days, included removal of external fixator and the conversion to stable fixation of the shaft fracture with retrograde i.m. nailing. The proximal interlocking of the retrograde nail can simultaneously be performed with the screws which are utilized for fixation of the DHS plate (Figure 1a,b).

The postoperative treatment included X-ray control of the femur using AP and lateral views with adjacent knee and hip joints. Physiotherapy was started on postoperative day 2, and weight bearing was limited to 10 kg partial weight bearing for 6 weeks. Consecutively, weight bearing was increased according to progress of bone healing. Regular follow-up visits including clinical and radiological studies at 6 week intervals were performed for 6 months. In terms of short-term follow-up, at 6 months, all but four patients were assessed during office visits. Four patients died and, thus, were lost to follow-up. During the second year, follow-up visits were extended to 3-month intervals. Bone healing was assessed radiologically and clinically using conventional X-ray studies, as well as by evaluating clinical symptoms including pain with full weight bearing. Healing was concluded with formation of bridging callus and bone trabeculae crossing the fracture line in at least three out of four cortices, as well as the absence of pain with full weight bearing. Axis alignment was assessed by analyzing digital X-ray images for varus and valgus axis deviation, as well as leg length discrepancy. Torsion was evaluated clinically by comparison of ipsi- and contralateral range of motion of hip joints. In patients with clinical signs conclusive for significant rotational axis deviation, rotational CT scan analysis was performed. The removal of hardware was performed electively, approximately 1 year after confirmation of bone healing.

Assessment of functional results was performed using the system of Friedman/Wyman including daily activities, range of motion (ROM) of hip and knee joints, return to work, return to sports activities as prior to the injury. Patients were evaluated for persistence of swelling, pain, and measurement of muscle circumference [5].

Statistical analysis was performed using SPSS® (SPSS, Chicago, Illinois, USA). Results in this study are presented as median values. Significance was statistically calculated based on Pearson’s chi-squared test. A result was considered to be statistically significant with *p*-value <0.05. Written informed consent was obtained from the patients for publication of this study and any accompanying images. Ethical clearance was obtained from Institutional Ethical Committee, and the study adhered to the tenets of the Helsinki Declaration of 1975, as revised in 2000.

## Results

**Figure 2:**
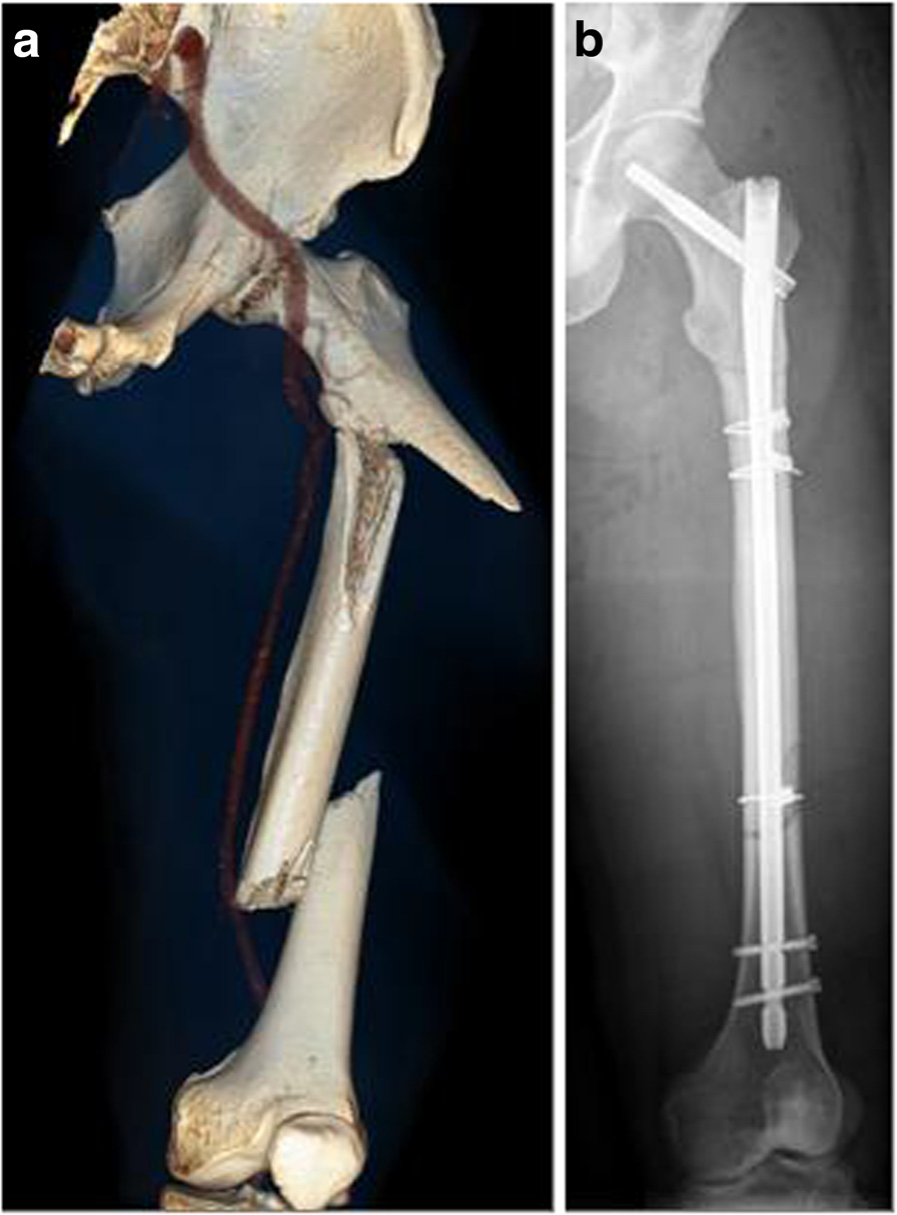
**41-year-old male patient after multiple trauma (unstable trochanteric fracture and shaft fracture. (a)** One-step stabilization with all-in-one device using a long cephalomedullary nail and fixation of the intermediate (floating) fragment with cerclages **(b)**.

**Figure 3:**
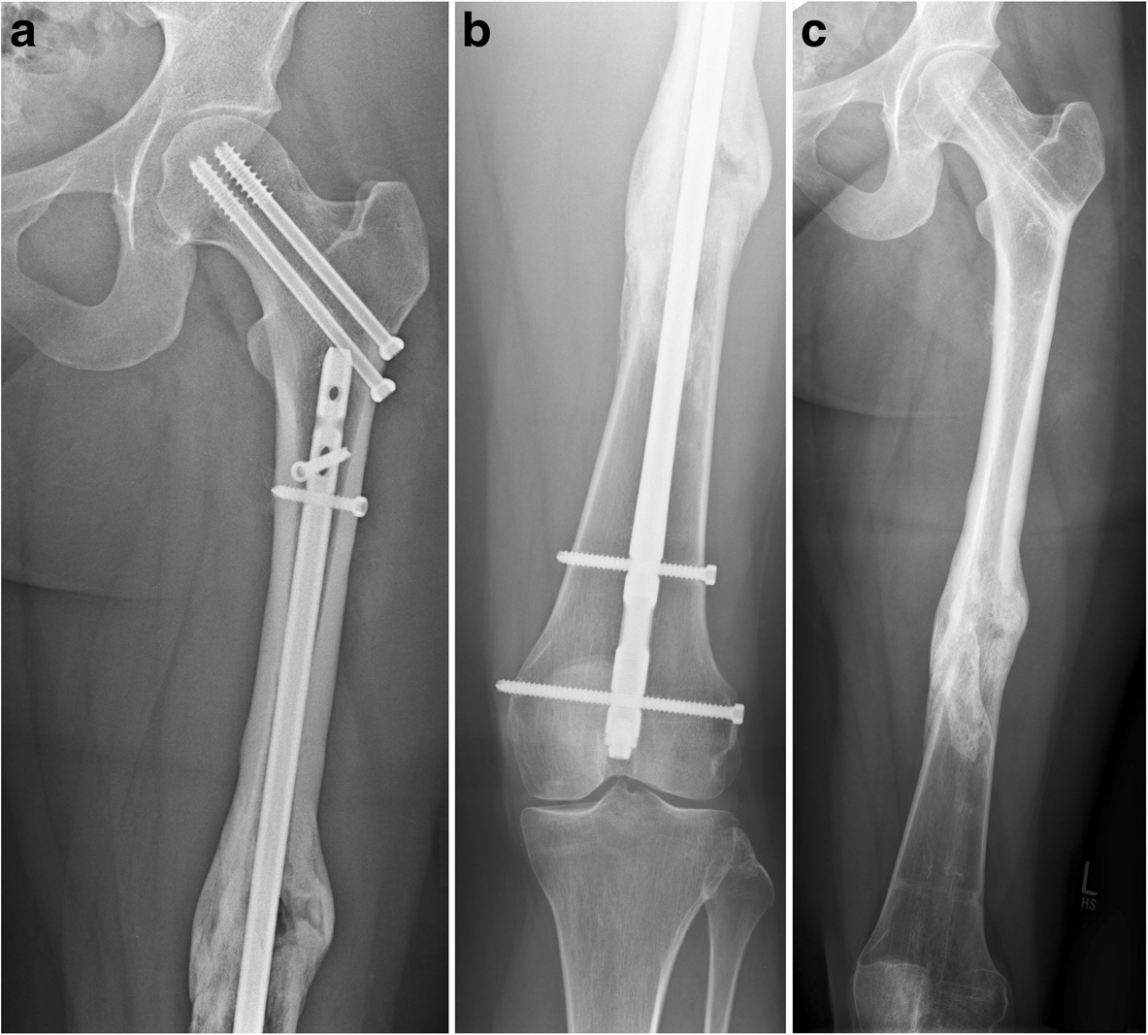
**28-year-old female patient with monotrauma: femoral neck fracture and distal shaft fracture.** Initial stabilization with retrograde nail and screw osteosynthesis **(a, b)**. Implant removal was performed 1 year after trauma after confirmation of complete bone healing **(c)**.

For osteosynthetic stabilization of multi-level femoral fractures, all-in-one device, e.g., cephalomedullary nail, was used in 36 patients, “rendezvous” technique using the combination of retrograde i.m. nailing and DHS osteosynthesis in 9 patients, and the conventional technique with two non-overlapping implants in the remaining 16 patients (Table 1). In 24 out of 65 patients, a two-step strategy was used to stabilize the fracture components whereas 36 patients were treated using a single step procedure. When using the two-step strategy, the staged exchange procedure was performed at an average of 5 days after the initial surgery. Four out of these 24 patients died prior to the second step of surgical procedure due to early multi-organ failure (3 patients) and severe traumatic brain injury (1 patient). Fracture stabilization with all-in-one device technique was performed using a long cephalomedullary nail in 14 patients (9 patients without and 5 patients with additional cerclage wiring; Figure 2a,b), a retrograde nail in 8 patients, and a proximal femoral nail in 14 patients (9 patients without and 2 patients with auxiliary anti-rotational plating). In the conventional group with two non-overlapping implants (16 patients), the following implant combinations were used: 4 patients DHS + locking plate osteosynthesis, 3 patients DHS + retrograde nail using non-overlapping implants, 2 patients single screws + retrograde nail (Figure 3a,b), 3 patients cephalomedullary nail + locking plate, 1 patient single screws + locking plate, 1 patient proximal femoral nail + locking plate, and 2 patients total hip arthroplasty + locking plate.

In the “rendezvous” group using the surgical technique of overlapping implants, eight patients were treated with DHS + retrograde nail (Figure 1a,b) and one patient with single screws + antegrade nail.

Fracture healing 2 years after trauma was observed in 57 out of the remaining 61 patients (“rendezvous”: 9, all-in-one device: 33, conventional:15; *p*-value: 0.66; Table 2) as assessed both radiologically and clinically. No significant difference concerning complication rates between the three cohort groups was detected (“rendezvous”: 3, all-in-one device: 13, conventional: 5; *p*-value: 0.94; Table 3).

**Table 2:** **Fracture healing 2 years after trauma**

**Implant**	**Fracture healing**	**No fracture healing**
All-in-one device (*n* = 36)	33	3
Conventional (*n* = 16)	15	1
“Rendezvous” (*n* = 9)	9	0

Chi-square test: *p*-value 0.66.

**Table 3:** **Complications following operative treatment**

**Implant**	**Complications**	**No complications**
All-in-one device (*n* = 36)	13	23
Conventional (*n* = 16)	5	11
“Rendezvous” (*n* = 9)	3	6

Chi-square test: *p*-value 0.94.

**Figure 4:**
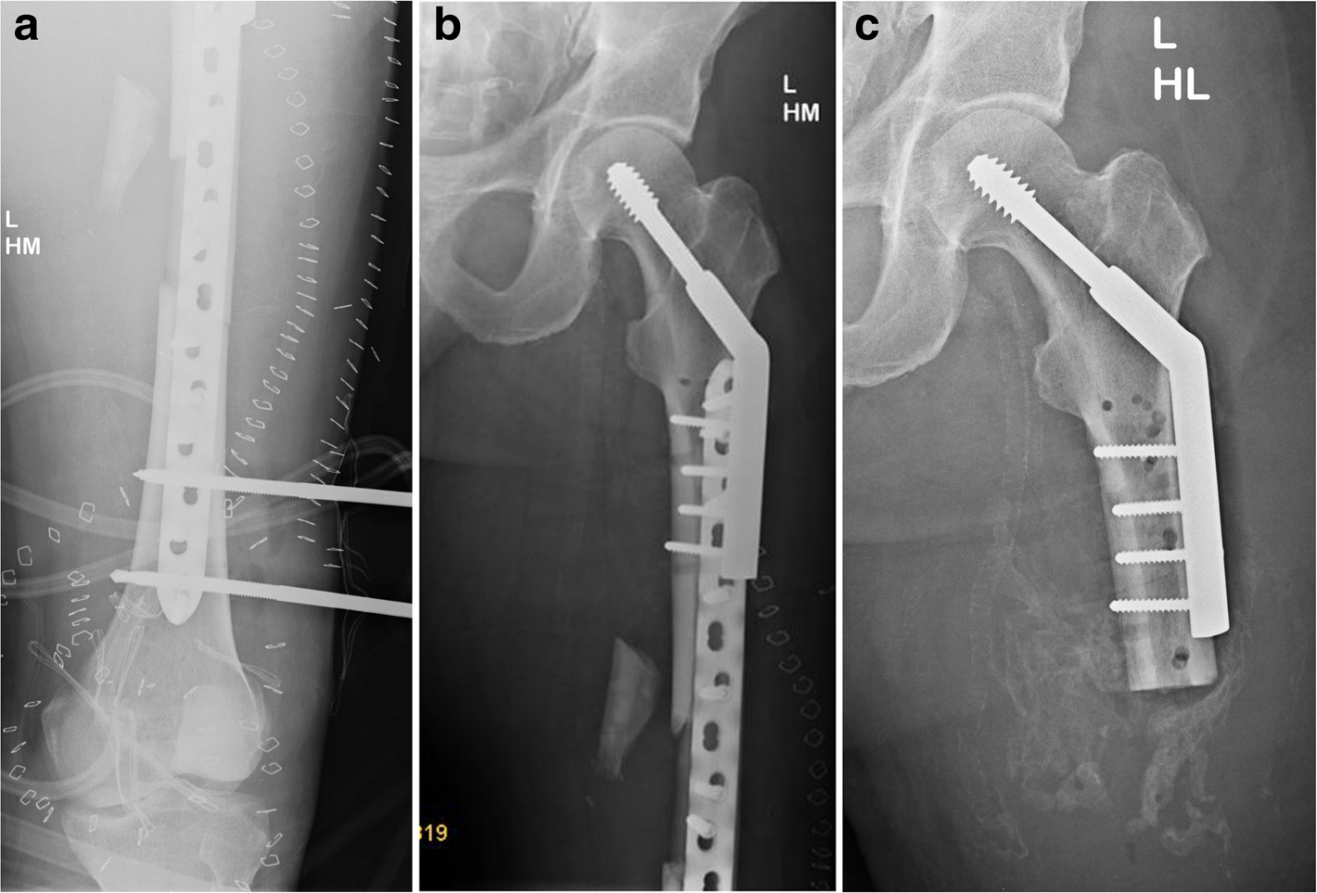
**48-year-old male patient who suffered a crush injury to his lower leg: femoral neck fracture and shaft fracture and knee dislocation with severe soft tissue damage including the popliteal artery.** Two-step strategy with initial treatment using external fixator and vacuum therapy and secondary definite fixation with DHS and locking plate **(a, b)**. Due to the artery lesion and fatal soft tissue damage of the lower leg, secondary amputation of the femoral shaft was necessary **(c)**.

The following complications were found: missed proximal fracture (two patients), implant failure (two patients: 1 × cephalomedullary nail, 1 × locking plate), deep infection (one patient), varus axis deviation of femoral neck (one patient) following screw osteosynthesis, and axis deviation of the femoral shaft (one patient) following retrograde nail osteosynthesis. In another patient, retrograde nailing was combined with cephalomedullary nail for the proximal fracture. In this patient, non-union of the shaft fracture was observed whereas the proximal fracture healed uneventfully. In another patient who suffered from a crush injury to his lower leg with damage of the popliteal artery resulting in femoral amputation, bone stabilization was performed using the combination of DHS osteosynthesis for the proximal fracture and locking plate fixation of the shaft fracture (Figure 4a,b,c).

The cohort group consisted of 65 patients. From the remaining 61 patients, 57 were assessed clinically and radiologically during office visits which were performed at our institution. Assessment of axis alignment following bone healing 24 months after trauma (range 13–42 months) demonstrated mean axis deviation for varus of 5° and for valgus of 5°. Comparison of leg length demonstrated a discrepancy of a mean of 1 cm of shortening for the injured side (range from 0.5 to 2.5 cm). Femoral torsion demonstrated axis deviation of 5° following surgery and complete bone healing. Assessment of functional results demonstrated ROM of the hip joint with a mean of 100° hip flexion and ROM of the knee joint with a mean of 110° flexion. No extension deficit for both the hip and knee joints was observed. Clinical long-term outcome 2 years after trauma, using the assessment system of Friedman/Wyman, is presented in Table 4 [5]: in the “rendezvous” group, 77.7% of patients had a good clinical long-term result, in the all-in-one device group 77.8%, and in the conventional group 75%. The majority of patients did not demonstrate persistence of swelling and pain. Elective removal of hardware was performed approximately 1 year after successful bone healing. In 28 patients, removal of implants has been already performed.

**Table 4:** **Long-term outcome according to the functional assessment system of Friedman/Wyman (Friedman and Wyman 1986)**

**Result**	**Impairment of ADL**	**Pain**	**Loss of hip or knee ROM (%)**
All-in-one device (*n* = 36)			
Good: 28	None: 30	None: 24	<20: 28
Fair: 6	Mild: 5	Mild/moderate: 10	20–50: 7
Poor: 2	Moderate: 1	Severe: 2	>50: 1
Conventional (*n* = 16)			
Good: 12	None: 11	None: 12	<20: 13
Fair: 2	Mild: 5	Mild/moderate: 4	20–50: 2
Poor: 2	Moderate: 0	Severe: 1	>50: 1
“Rendezvous” (*n* = 9)			
Good: 7	None: 8	None: 7	<20: 8
Fair: 2	Mild: 1	Mild/moderate: 2	20–50: 1
Poor: 0	Moderate: 0	Severe: 0	>50: 0

*ADL* activities of daily living, *ROM* range of motion.

## Discussion

Ipsilateral proximal and shaft fractures of the femur are caused by high velocity or high energy trauma with force transmission along the axis of the femoral shaft. Force impaction usually occurs on the femoral shaft and causes femoral fracture typically in the middle third. Forces progress to the proximal femoral region and often result in a vertical fracture line at the baso-cervical junction without dislocation of fracture fragments. Shuler analyzed 52 patients and reported a predominance of femoral neck fractures (90%) compared to trochanteric fractures (10%) in the proximal femur fracture [6]. Table 5 summarizes possible fracture combinations of the specific entity of proximal femoral fractures. However, for surgical assessment and surgical strategy, it is critical to understand that the fragment between the proximal fracture and the shaft fracture is the so-called intermediate fragment. Lin et al. considered this intermediate fragment the key fragment of the fracture and called it the “floating” fragment which required stable fixation and, more specifically, rotational stability [7]. Regarding the surgical strategy, both fracture components, the proximal and the shaft fracture, require anatomic reduction and anatomic realignment of axis and torsion. At the proximal fracture site, a moderate valgus position of the proximal fragment following fracture reduction is acceptable. In contrast, any varus axis deviation of the proximal fragment should be avoided. In order to avoid varus axis deviation, it may be necessary to use an open approach. For successful bone healing, stable fixation of both fractures is required.

**Table 5:** **Possible fracture combinations of the specific entity of proximal femoral fracture**

	**Screw**	**DHS**	**AN**	**RN**	**LP**	**CN**
Proximal fracture						
Medial FNF undisplaced	+	+				−
Medial FNF displaced	(+)	+				−
Lateral FNF	−	+				−
Stable trochanteric fracture	−	+				(+)
Unstable trochanteric fracture	−	−				+
Subtrochanteric fracture	−	−				+
Distal fracture						
Shaft fracture three fifth			+	(+)	(+)	+
Shaft fracture four fifth			+	+	+	−
Distal fracture without joint involvement			−	+	+	−
Distal fracture with joint involvement			−	+	+	−

*Screw* screw fixation, *DHS* dynamic hip screw, *AN* conventional anterograde nail, *RN* retrograde nail, *LP* locking plate, *CN* cephalomedullary nail, *FNF* femoral neck fracture, *+* good indication, *(+)* limited indication, *−* not indicated.

The surgical fracture stabilization is considered an emergency procedure. Analogous to the treatment of all multi-level limb fracture, it is recommended to proceed with fracture stabilization from distally to proximally. If the femoral neck fracture is displaced, the surgical strategy of choice is first to stabilize the shaft fracture followed by screw fixation of the proximal fracture. The stabilized shaft fracture facilitates reduction of the dislocated proximal fracture. This surgical strategy facilitates reduction and results in improved alignment of both fracture components.

In case of polytraumatization, temporary stabilization of the distal fracture using an external fixator can be achieved. After that, we proceed with fracture stabilization of the proximal fracture using either screw fixation or DHS osteosynthesis. Extension of the external fixator to the pelvic ring to include the proximal fracture is possible but stabilization of the proximal fracture with this temporary fixation is insufficient.

For treatment of the multi-level femur fractures, the single-step or step-wise procedures have specific indications:

1. *Staged procedure in multiple trauma patients*
  - The first procedure includes stabilization of the distal shaft fracture using external fixator followed by closed or open reduction and internal fixation of the proximal fracture.
  - Definitive stabilization of the shaft fracture is performed electively following amelioration of the general conditions of the patient.

Advantages of step-wise stabilization of both fracture components include:

1. Easy fracture stabilization, specifically in treatment of polytraumatized patients
2. Reduction of fractures step by step
3. Facilitation of fracture reduction proximally following stable fixation of fractures distally
4. Reduced operation time on the day of injury according to the concept of DCO for polytrauma treatment [8]

2. *Single-step procedure in monotrauma patients*
  If the proximal fracture is displaced, it is recommended to start with stabilization of the distal fracture using internal fixation devices followed by stabilization of the proximal fracture. Only if the distal or shaft fracture is stabilized, reduction maneuvers for closed reduction of the proximal fracture can be employed. If the proximal fracture is not displaced, the surgical procedure may be started with stabilization of the proximal fracture to avoid secondary dislocation intraoperatively. In case of a non-displaced proximal fracture, the principles of multi-level fracture treatment with distal to proximal stabilization strategy may be neglected.

### Fracture stabilization using all-in-one device vs. implant combinations

Multi-level femoral fractures can be treated using an all-in-one device, e.g., a long cephalomedullary nail (Figure 2a,b). Originally, the idea of stabilizing the ipsilateral proximal (femoral neck or trochanteric fracture) and the femoral shaft fracture using all-in-one device implants was intriguing [9]. The disadvantage of this treatment concept is the relatively unstable implant. Regularly, thin cephalomedullary nails are inserted which do not exert a snug fit in the intramedullary canal. Due to the limited options for inserting interlocking screws, rotational stability is not granted. Specifically, the intermediate fragment is not sufficiently stabilized. Lin et al. have introduced the concept of the so-called floating fragment which is extending from the greater trochanter to the shaft fracture and which is not sufficiently stabilized, neither proximally nor distally. Additional rotational stability of the intermediate fragment can be achieved by insertion of cables via a minimally invasive approach and utilization of a special cable clamp. Prerequisite for additional cable fixation is an oblique or spiral fracture configuration [7,10,11]. Alternatively, some authors suggest application of an auxiliary plate osteosynthesis with monocortical screw fixation to increase rotational stability of the intermediate fracture fragment [12,13].

### Combination of implants

Indications for screw fixation of proximal fractures include stabilization of non- or minimally displaced medial femoral neck fractures [14]. Typically, three 6.5-mm lag screws (cannulated if necessary) are inserted. Screw fixation can be combined with retrograde or anterograde nailing of the shaft fracture.

Conventional anterograde nailing is the gold standard for treatment of fractures in the three or four fifth of the femoral shaft combined with a femoral neck fracture [15]. Anterograde nailing can be combined with screw fixation of the femoral neck fracture using the miss-a-nail technique in a single-step surgical strategy. Some authors have reported to apply specific aiming devices for this technique [4]. Consequently, anterograde nailing has to precede insertion of screws. In non-displaced femoral neck fractures, it is possible to start fracture treatment with stabilization of the proximal fracture using screws osteosynthesis. The lag screws secure the femoral neck fracture and prevent secondary fracture dislocation. However, this surgical strategy requires retrograde nailing of the shaft fracture (Figure 3a,b,c).

Retrograde nailing is indicated for stabilization of distal femoral fractures including knee joint involvement as well as shaft fracture located in the distal 4th and 5th fifth of the femoral shaft and irrespective of type of proximal fracture fixation using either screw fixation or DHS osteosynthesis [16]. In addition, retrograde nailing offers the possibility to apply the so-called “rendezvous” surgical technique with step-wise and staged fracture stabilization including primary treatment on the day of injury using external fixator for the shaft fracture and definitive internal osteosynthesis for the proximal fracture. In a second step, which is performed following stabilization of the general conditions of the patient after several days, the external fixator is removed and the shaft fracture is stabilized using retrograde i.m. nailing. The retrograde nail offers the possibility for dynamic compression of the shaft fracture and can be easily combined with DHS osteosynthesis using the "rendezvous" technique. The lateral screws which are utilized for plate fixation of the DHS device simultaneously serve as interlocking screws for the retrograde nail (Figure 1a,b). In addition, the AP interlocking screw of the nail is not compromised and secure stabilization of both fractures components is ensured.

Indications for osteosynthesis using the DHS device include femoral neck and stable trochanteric fractures which demonstrate intact medial buttress. In displaced and non-displaced femoral neck fractures, DHS osteosynthesis is advantageous by providing stable fixation specifically if fracture line is proceeding laterally or baso-cervically. DHS osteosynthesis can be performed as a first step procedure in non-displaced proximal fractures. In displaced fractures, it is recommended as described above to first stabilize the shaft fracture and secondarily the proximal fracture. In femoral neck fractures, insertion of an additional, anti-rotational lag screw is mandatory to increase rotational stability,to preferentially achieve a discrete valgus position of the femoral head fragment and to avoid detrimental spinning of the femoral head fragment, while inserting the DHS femoral neck screw.

Alternatively, plate osteosynthesis of a shaft fracture can be performed if the proximal fracture has been stabilized using screw fixation [17]. Exceptionally, plate osteosynthesis may also be combined with a DHS implant if distally to the DHS plate at least three to four plate holes can be placed into the proximal shaft fragment. Plate osteosynthesis is predominately indicated in specific conditions such as increased risk for infection, pulmonary impairment, or distal limb amputation (Figure 4a,b,c).

## Conclusions

The anatomic fracture configuration can be categorized into specific fracture types both proximally and distally. According to the fracture composition, various implant combinations for fracture stabilization may be selected. Typically, the distal fracture is the “leading” fracture component. Possible implant combinations include screw fixation proximally and anterograde or retrograde nailing as well as plate fixation of the shaft. Alternatively, DHS osteosynthesis proximally can be combined with retrograde nailing or plate fixation distally using the so-called “rendezvous” surgical technique.

In cases with unstable trochanteric and shaft fracture, or subtrochanteric multi-level fractures, the proximal fracture is considered the “leading” fracture. For these fracture configurations, stabilization using all-in-one device implants such as the cephalomedullary nail is recommended. Fractures, which are localized very distally are best treated with retrograde i.m. nailing.

In polytrauma patients, the DCO concept mandates short operation time on the day of injury, followed by definitive fracture treatment in the interval after patient recovery. Thus, the staged “rendezvous” technique is a beneficial treatment concept for these patients.

### Consent statement

Written informed consent was obtained from the patients for publication of this report and accompanying images. A copy of the written consent is available for review by the Editor-in-Chief of this journal.
